# Supplementary material for: Differences in mediolateral dynamic stability during gait initiation according to whether the non-paretic or paretic leg is used as the leading limb
Source: PLoS One. 2022 Apr 27;17(4):e0267577. doi: 10.1371/journal.pone.0267577 (PMC9045617; doi:10.1371/journal.pone.0267577)
Supplement: Attachment — Submitted filename: PONE-D-21-29688_Review.pdf [file pone.0267577.s002.pdf]

Manuscript Review

Title

Differences in the use of the leading limb in gait initiation in individuals with stroke

General Comments

1. The authors' intended meaning of dynamic stability is not clearly defined and I believe is a point of confusion throughout the manuscript as they also mention balance control, particularly in the discussion. Being in a stable position is not the same as being able to control that position. Whether the paretic or non-paretic limb should be used to initiate gait would be dependent on a combination of these factors: in which configuration (paretic vs. non-paretic leading limb) are they most stable and in which configuration do they have the best ability to control their balance? This important distinction and consideration is not addressed by the authors, which I believe leads to the contradictions noted in the discussion (see next comment).
2. In general, the discussion tends to contradict the authors' conclusion more than it supports their conclusion (see specific comments 16-18). This is a major concern which suggests that the authors are not drawing appropriate conclusions from their data.
3. The methods mention the subjects had a favored leading limb and that this limb was always used first in the task. However, it is not clarified whether participants "favored" their paretic or non-paretic limb and how this might have affected their findings. How did results differ between the favored and unfavored limb as compared to the paretic and non-paretic limb? Which limb did the participants select and how did this compare to their biomechanics based on your metrics?
4. Please put citations *inside* punctuation. This greatly improves readability of the manuscript.

Specific Comments

Introduction

1. Line 39: The authors state that posture is unconsciously controlled during walking; however, it would be more accurate to state that posture is unconsciously controlled during "*steady-state*" or "*unperturbed*" walking.
2. Lines 49-51: The interpretation of the findings of reference 5 seem to be overstated by the authors. While the article does suggest that the use of the paretic leg as the leading leg is associated with difficulties activating the tibialis anterior and gluteus medius muscles during the anticipatory postural adjustment phase of gait initiation, the same article also states "In contrast, the use of the paretic leg as the trailing leg seems to challenge balance to a greater extent during GI" and in their conclusion "GI is facilitated when the non-paretic leg is used as the trailing leg because the weakness of the paretic leg leads to difficulties in supporting body weight during the upcoming stance phase." Please accurately present the findings of the previous study in the introduction.
3. Lines 54-57: This sentence requires some clarification. First, what were the different evaluation indices used in each study? Second, it is unclear what the conclusions of references 7 and 8 (Tokuno and Brunt, respectively) found with regards to which leading limb is preferable.
4. Line 61: Statement should read "However, center of pressure *displacement*…"

5. Lines 62-64: Please elaborate on why the use of the extrapolated center of mass/margin of stability is a superior measure of dynamic balance than center of pressure displacement.
6. Lines 67-69: Please more clearly define the purpose statement. As written, the purpose is unnecessarily wordy.

Methods

7. Lines 82-83: Please explicitly state the university and hospital which gave approval for this study. Also, this sentence does not need to be its own paragraph.
8. Lines 96-98: Several issues need to be addressed: What is meant by "favored" leading limb and how was this determined? Did the participant's preference of favored limb influence any of the outcome measures? What are the potential limitations of not randomizing the initial leading limb? Was the initial stance width controlled? How might the initial stance width influence the MOS?
9. Lines 103-104: Given the importance of accurately identifying the center of mass location in your primary outcome measure, the calculation of the center of mass location should be more fully explained.
10. Lines 127-128: What is the purpose of the "additional indices" used in the study? Why were these three selected? What additional information do they provide?
11. Lines 128-132: It would be helpful to number the 3 phases (e.g., 1) the postural phase, …; 2) the monopodal phase…).
12. Lines 134-135: The calculation of the lateral pelvic tilt angle is not sufficiently explained. What markers were used?
13. In Figure 3, the time is defined from 0-200%; however, nowhere in the methods is the time normalization of the data explained. Please define these methods.

Results

14. While p-values/effect sizes are presented in Table 2, it would be helpful to have these data included in the text. Moreover, the authors should provide a metric of how much larger/smaller (raw difference or percent difference between averages/medians) a variable was for a given condition when describing their results.

Discussion

15. Lines 183-184: In which condition did the %MoS exceed 100% in more than half of the participants? Please clarify.
16. Lines 184-186: The statement about negative behaviors associated with paretic limb loading seem to contradict the authors' assertion that the non-paretic leading limb (paretic trailing-limb) is better.
17. Lines 188-190: The statement that a large MOS in the lateral direction indicated poor ability to maintain balance appears to contradict the author's assertion that the non-paretic leading limb is better based on their findings that a larger MOS was observed with the non-paretic leading limb.
18. Lines 203-204: Again, this statement that individuals with stroke may find it easier to used the paretic limb as the leading limb contradicts their conclusion.

Tables and Figures

1. Figure 3: The standard deviation of the non-paretic limb is hard to distinguish from that of the paretic limb (it’s unclear where the lower bound of the standard deviation of the non-paretic limb is).
